# Accumulation of Ascorbic Acid in Tomato Cell Culture: Influence of the Genotype, Source Explant and Time of In Vitro Cultivation

**DOI:** 10.3390/antiox9030222

**Published:** 2020-03-07

**Authors:** Maria Minutolo, Pasquale Chiaiese, Antonio Di Matteo, Angela Errico, Giandomenico Corrado

**Affiliations:** Dipartimento di Agraria, Università degli Studi di Napoli “Federico II”, 80055 Portici, Italy; minutolm@unina.it (M.M.); adimatte@unina.it (A.D.M.); errico@unina.it (A.E.); giacorra@unina.it (G.C.)

**Keywords:** vitamin C, *Solanum lycopersicum*, *Solanum pennelli*, Introgression Lines, in vitro tissue culture, calli, gene expression

## Abstract

The production and commercialization of natural antioxidants is gaining increasing importance due to their wide range of biological effects and applications. In vitro cell culture is a valuable source of plant bioactive compounds, especially those highly dependent on environmental factors. Nonetheless, research on the accumulation in plant cultured cells of water-soluble antioxidant vitamins, such as the ascorbic acid (AsA), is very limited. Tomato fruits are a main dietary source of vitamin C and in this work, we explored the potential of in vitro cultured cells for AsA accumulation. Specifically, using a full factorial design, we examined the effect of the source explant, the time in tissue culture and the genetic difference present in two Introgression Line (IL7-3 and IL12-4) that harbor Quantitative Trait Loci (QTLs) for ascorbic acid in fruits. Moreover, we performed an expression analysis of genes involved in AsA metabolism to highlight the molecular mechanisms that can account for the difference between fruit explants and calli. Our work indicated that cultured tomato cells accumulate AsA well beyond the amount present in fruits and that the three factors under investigation and their interaction significantly influence AsA accumulation. The time in tissue culture is the main single factor and, different from the expectations for secondary metabolites, explants from unripe, mature green fruits provided the highest increase in AsA. Moreover, in controlled conditions the genetic differences between the ILs and the control genotype are less relevant for calli cultivated for longer time. Our work showed the potential of tomato cell culture to produce AsA and prompt further refinements towards its possible large-scale exploitation.

## 1. Introduction

Ascorbic acid (AsA), also indicated with the broader term “vitamin C”, is an essential nutrient for humans [1]. AsA is a cofactor in different enzymatic reactions and participates in a variety of biological functions, both in mammals and plants [1]. In the human diet, AsA has gained popularity as antioxidant especially for its ability to counteract the over-production of free radicals in cells, and as a natural chemopreventive for the reduction of risk factors for cardiovascular diseases and cancer [2,3,4].

Fruits and vegetables are the main dietary source of antioxidants [5]. After oranges, tomatoes (*Solanum lycopersicum* L.) are the most important source of vitamin C [6], also because they are often eaten raw and in relatively large quantity compared to other spices, fruits and aromatic herbs. AsA accumulation in tomato and in other plant species is a complex process that involve different metabolic pathways [7]. In higher plants, compelling evidence for AsA *de novo* synthesis has been provided essentially for the d-mannose/l-galactose pathway [8,9]. Additional pathways are based on the conversion of 1-d-myo-inositol, d-galacturonic acid or l-gulose, although the l-gulose pathway is also considered a branch of the l-galactose pathway [10]. AsA accumulation also depends on recycling and degradation as well as sink-source relations [9,11]. The quantitative variation of AsA in edible organs is a heritable trait with a complex genetic basis, also because of the number of genes that can directly or indirectly influence its metabolism. A known source of trait variation within a plant species is represented by genetic factors [12]. In various trees and horticultural species, varieties differ in the amount of ascorbic acid in edible organs [13,14,15]. Moreover, a range of endogenous and external factors, including phenological, physiological and environmental cues, can have a significant effect on the accumulation of AsA in the different plant tissues [12].

Although tomato fruits are considered a good dietary source of AsA, cultivated varieties typically have a lower percentage of AsA than *S. lycopersicum* wild relatives, such as *S. pennellii* [16]. This difference has been exploited for the QTL mapping of the AsA content and other quantitative parameters of tomato fruits [16,17,18,19]. These activities have been carried out by exploiting Introgression Lines (ILs). These are *S. lycopersicum* lines that contain a fraction of the genetic material of a wild relative, introduced by crossing followed by repeated backcrossing and DNA markers-selection. A widely used IL-population derived from crossing the cultivated variety M82 with *S. pennelli* [20]. Different studies identified two lines (IL7-3 and IL12-4) that harbour QTLs for AsA accumulation [16,21,22,23]. Although ILs are an important experimental tool in genetics and breeding, they are not designed to be cultivated [24]. For instance, IL7-3 also has a negative QTL for yield [20].

Antioxidants usually occur at low concentrations in plants [6]. Moreover, AsA content decreases with the storage time of the fresh vegetable [25,26]. To satisfy the commercial requirements, vitamin C is chemically synthesized using industrial methods based on the Reichstein process. In recent years, greater attention has been given to antioxidants from natural sources as alternative to synthetic compounds created from chemical processes [27]. The market of natural antioxidants is expected to increase in the near future for pharmaceuticals, to replace synthetic antioxidants as a food additive (e.g., in meat, as browning inhibitor, flavor and color stabilizer, etc.), and for cosmetics [28,29,30]. Plant cell culture represents an interesting option for the controlled and scalable production of natural bioactive metabolites, especially those strongly dependent on a variety of environmental factors and/or accumulating in fruits or organs at late stage of cultivation [31,32]. Explants from different plant tissues, in appropriate conditions, can generate a cell mass (i.e., the callus) that can grow in vitro indefinitely. Moreover, a callus can be used to yield cell suspension cultures that are amenable to bioreactor scale-up and ultimately, industrial production [33,34]. The possibility for a cost-effective production of natural bioactive compounds by callus cultures has been exploited for different medicinal and horticultural plants, as recently reviewed [32,35].

In this work we evaluated the potential of available tomato ILs to produce AsA in vitro. Specifically, our aim was of investigating the effect of main factors (i.e., genotype, stage of the source organ, time in tissue culture) and their interaction on the quantitative variation of ascorbic acid in plant tissue culture. Moreover, we integrated our analysis with an investigation of the expression level of genes involved in the biosynthesis and recycling of AsA in tomato. This analysis also allowed to underline the role of the expression level of different genes of the AsA pathways in controlled conditions.

## 2. Materials and Methods

### 2.1. Plant Material and Tissues Culture

Seeds of the *S. pennellii* introgression line 7-3 (Acc. LA4066) and 12-4 (Acc. LA4102) [20], and their background genotype *Solanum lycopersicum* ‘M82’ (Acc. LA3475) were provided by the Rick Tomato Genetics Resource Center (Davis, CA, USA). Plants grew in a greenhouse in a mixture (1:1 *v*/*v*) of volcanic sandy soil and commercial substrate (Professional Substrate Type-S, FloraGard, (Oldenburg, Germany)) with standard cultural practices (e.g., watering and fertilization). Fruits were harvested at three stages: mature green (MG), turning red (TR) and red ripe (RR). For tissue culture experiments, fruits were surface sterilized with a solution of 70% ethanol for two minutes (min), then left for 15 min in a solution of 2% sodium hypochlorite and 0.1% (*v*/*v*) Tween-20. Finally, fruits were rinsed in sterile distilled water for three times. We employed a full factorial experimental design. For each experimental thesis, explants (approximately 1 cm^2^ square face) were obtained from fruit pericarp and cultivated in Petri dishes containing 20 mL of MSTC medium solidified with 0.8% (*w*/*v*) of agar sealed with Parafilm M. The MSTC medium (pH 5.8) comprised MS salts [36] supplemented with of 30 gL^−1^ sucrose, 2 μM of 2,4-Dichlorophenoxyacetic acid (2,4-D) and 1 μM of 6-Benzil-ammino-purine (BAP). Chemicals were purchased from Duchefa Biochimie (Amsterdam The Netherlands). Explants were incubated at 24 ± 2 °C under a 16/8 h (light/dark) photoperiod with a light intensity of 100 μmolm^−2^s^−1^. After initiation, calli were transferred to fresh medium every fifteen days. The callus induction frequency (CI) was calculated by the following formula:

(1) $$CI\;\left(\%\right)=\frac{\text{Number of explants induced callus}}{\text{Total number of explants cultivated}}\times 100$$

### 2.2. Ascorbic Acid Extraction and Quantification

The ascorbic acid content was estimated on fruit pericarp and after 15, 30, 45 and 60 days of in vitro culture from ten randomly chosen calli for each experimental thesis. Samples were taken during the first hours of the light period, weighted, frozen in liquid nitrogen and stored at −80 °C. Ascorbic acid was extracted as reported by [23] and it was measured as previously described [13,37]. AsA quantities were determined by subtracting the reduced AsA (rAsA) from the total Asa (tAsA).

### 2.3. RNA Isolation and Real-Time Quantitative RT-PCR Analysis

Total RNA was isolated from pericarp of mature green fruits and deriving calli (after 60 days of in vitro culture) from three biological replicates per genotype. Tissues were immediately frozen in liquid nitrogen and stored at −80 °C until use. Frozen tissues (approximately 100 mg) were homogenized with a TissueLyser (Qiagen, Milan, Italy) according to the manufacturer’s instructions. Total RNA was isolated and quantified as described [13]. First strand cDNA was synthesized using 1 µg of total RNA using the RevertAid First Strand cDNA Synthesis Kit (ThermoFisher Italia, Milan, Italy) according to the manufacturer’s guidelines. Real-time PCRs were performed in a 7900HT Fast Real-Time PCR System (Applied Biosystems, Milan, Italy), employing the Sybr Green chemistry. Amplifications were assembled in a final volume of 12.5 µL using the Power SYBR Green PCR Master Mix (Applied Biosystems, Milan, Italy) in technical duplicates per biological replicate. Primers are listed as Supplementary Materials (Table S1) and were checked for PCR efficiency (>1.8) as described [38].

### 2.4. Statistical Analysis

For callus induction, statistical analysis was performed with a Chi-square test and data are presented as percentage. For AsA measurements, data are expressed as mean ± standard deviation (SD). Statistical analysis was performed using a three-way between-subjectanalysis of variance (ANOVA), followed by post-hoc analysis with the Duncan’s multiple range-test (α < 0.05) considering as fixed factors the genotype (three levels), the time in tissue culture (five levels) and the maturity stage of the fruit explants (three levels). The homogeneity of variances over subpopulations was tested with the Levene’s test. An estimation of the effect size was carried out calculating the partial eta squared. The interpretation of the effect size values was performed as suggested [39,40]. Gene expression analysis was based on the relative quantification by the DeltaDeltaCt method [41] using the Elongation Factor 1-alpha as reference gene and the plant explant as calibrator genotype. The statistical analysis was performed using the Statistical Package for Social Sciences (SPSS 20) software version 20 (IBM Corp., Armonk, NY, USA).

## 3. Results

### 3.1. Morphogenesis Callus Response

**Figure 1:**
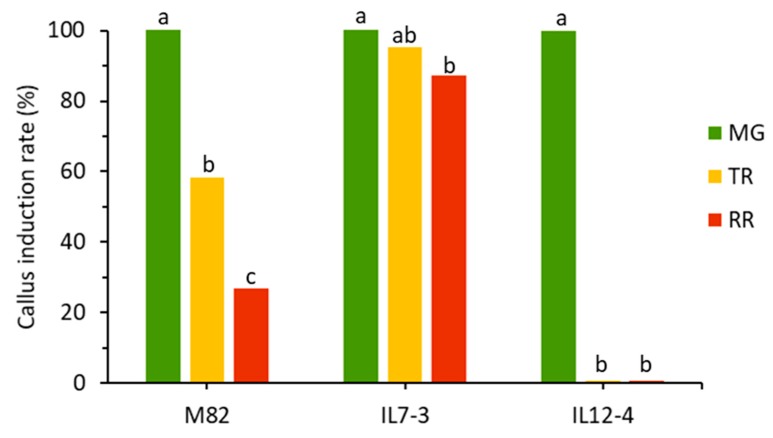
Callus induction frequency of the tomato genotypes. Explants of fruits at three maturity stage (MG: mature green; TR: turning red; RR: red ripe) were cultured on callus induction medium and the frequency of explants showing callus was determined after 15 days. For each genotype, different letters (a–c) represent statistically significant differences (*p* ≤ 0.05) among fruit explants.

The ability of *S. lycopersicum* cv M82 and two introgression lines (IL7-3 and IL12-4) to produce callus from fruits was evaluated at three development stages, namely mature green (MG), turning red (TR) and red ripe (RR). The two plant growth regulators (i.e., 2,4-D and BAP) induced a morphological modification of tomato fruits explants. The increase in thickness of explants was detected after few days of cultivation. After seven days, small calli were visible from the cut ends of the explants of all genotypes and types of explant. MG explants proved to be the best tissue for callus culture establishment for all genotypes investigated, with a CI frequency similar among the three genotypes. The same hormonal combination was generally less efficient on TR and RR explants after 15 days of culture. In relative terms, there was a significant difference among the genotypes in the ability to form callus according to ripening stage of the fruit (Figure 1). The IL7-3 showed a little reduction in the callus formation rate for fruit tissues taken at later maturity stage. For M82, the callus formation almost halved in relative terms from MG to TR, and from TR to RR. The largest difference was observed for IL12-4, which showed a very strong relative reduction in the callus induction considering explants from the TR and RR stages.

### 3.2. Ascorbic Acid Accumulation

To test whether the genotypes under investigation accumulate different amount ascorbic acid in controlled tissue culture conditions, ascorbic acid (AsA) and reduced AsA (rAsA) were quantified every 15 days in calli deriving from explant obtained at three development stages of tomato fruits (MG, TR, RR). Three-way analysis of variance (ANOVA) was used to evaluate the significance of the main factors (i.e., the genotype (Gen), the maturity of the fruit (Mat) and the age of the callus(Time)) and their interaction as source of variance for the amount of AsA. The statistical analysis indicated that there was a significant main effect of the Time, Genotype or Maturity on AsA (Table 1). Moreover, there was a significant three-way interaction among the factors.

The data indicate that the genetic differences among the two ILs and the ‘M82’ variety not only have a significant effect on AsA accumulation, but also that the time in tissue culture and the stage of the explant act together with the source genotype. Considering the two-way interaction between factors, the statistics indicated that there was a significant interaction effect between the pairwise combinations. Specifically, the time in tissue culture of the callus affected the accumulation of AsA in different ways in the three genotypes and the three fruit explants. Considering the percentage of variance of the AsA attributable to the different factors and their interactions, the Time * Mat interaction had the higher partial eta-squared, followed by the three-way interaction among factors. Another large effect size was that of the factor Time, while other factors and interactions had medium or small effect size. This is partially due to the presence of different significant interactions and a large sample population, but it also suggests a reduced linearity between the AsA response and the factors under investigation.

**Figure 2:**
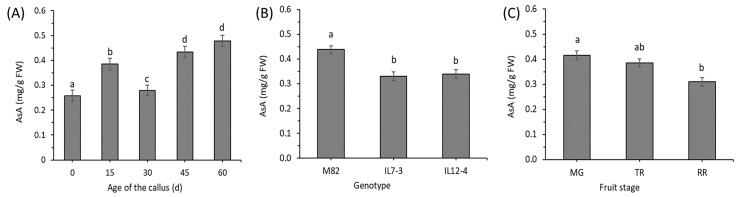
Main effect of the age of the callus (**A**), genotype (**B**) and stage of the fruit used for the tissue culture explants (**C**) on the AsA content. For each panel, different letters (a–d) represent statistically significant differences (*p* ≤ 0.05).

Although the interactions between factors imposes limitations in the interpretation of the single factor’s main effect, post-hoc analysis of the AsA during Time indicated that the age of the callus in tissue culture statistically increased the mean AsA content (Figure 2A). Specifically, the AsA content reached the maximum value (more than a 2-fold increase) after sixty days of in vitro growing. There was a statistical difference between the ‘M82’ variety and the two ILs (Figure 2B). Finally, also the stage of the fruit used as explant statistically affected the amount of the mean AsA. Specifically, the AsA content was lower for explants taken after the MG stage (Figure 2C).

### 3.3. Ascorbic Acid Dynamics in Tissue Culture

**Figure 3:**
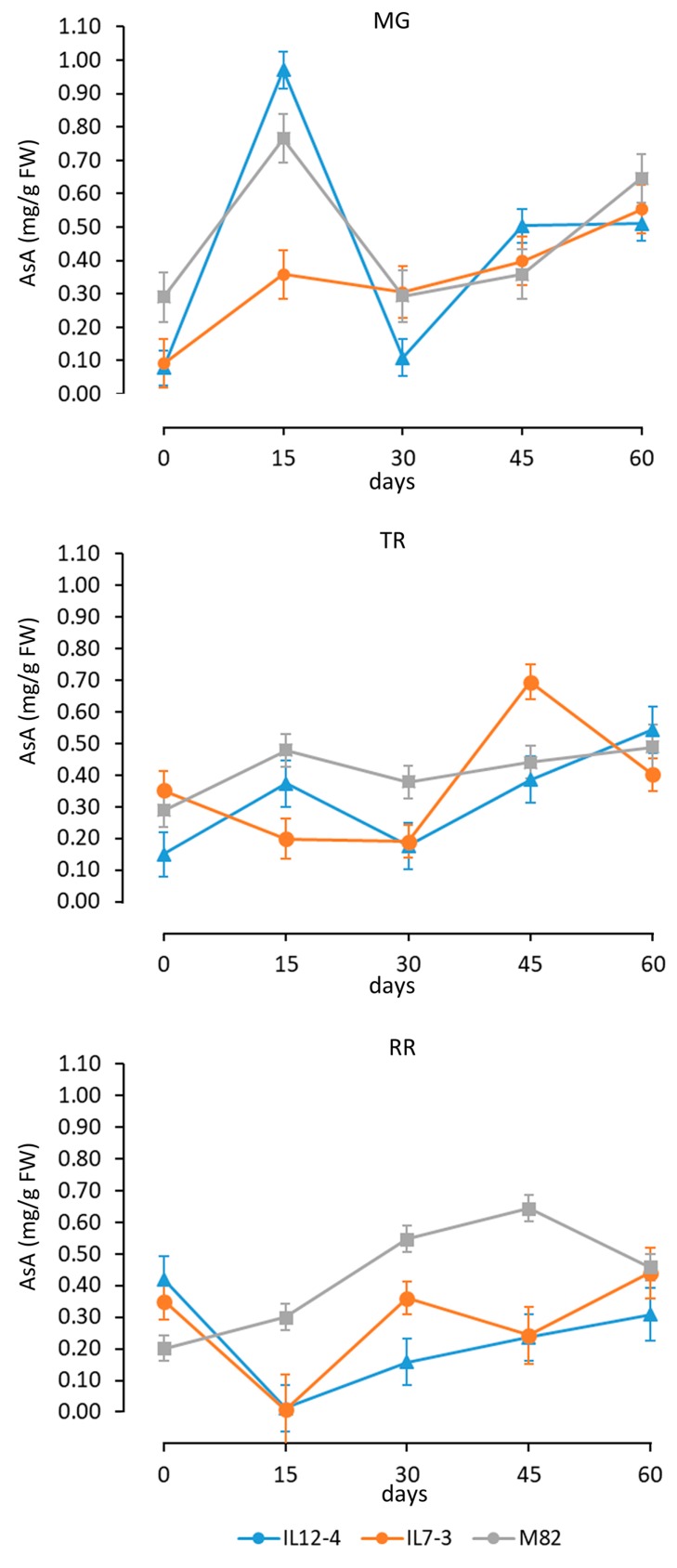
Dynamics of the ascorbic acid (AsA) in calli deriving from explant of fruits at the mature green (MG), turning (TR), and red ripe (RR) stage. For each source of explants, the graph reports the mean and standard deviation in the different genotypes from 0 to 60 days of tissue culture.

Considering that the two-way interaction between Gen and Mat had the lowest eta squared, we present the dynamics of the AsA accumulation during callus growth in relation to the different sources of explants (fruits at the MG, TR or RR stage) (Figure 3).

For explants from mature green fruits, the AsA amount strongly increased (approx. threefold) after 15 days in tissue culture for all three genotypes. After a decline (30 days), AsA increased reaching at the end of our analysis a similar amount in the three genotypes. In absolute term, the maximum AsA content was reached by IL12-4.

For explants from turning red tomatoes, the interaction between time and genotype was more evident. While the IL12-4 and M82 had a similar accumulation trends, the IL7-3 showed first a reduction, and then a peak at 45 days. Also for this time of explants, all the three genotypes reached similar AsA values at 60 days. For explants of red ripe fruits, the two ILs had a similar behavior. As expected, the AsA amount was at the RR stage higher than the control variety ‘M82’, and after an early decline (15 days), it increased with time. For the M82, the AsA content reached the maximum value at 45 days. Similarly, there was not a significant difference among genotypes at 60 days for the RR explants.

### 3.4. Reduced-AsA Accumulation

The three-way ANOVA indicated that there was a significant main effect of each of the three factors (i.e., the genotype (Gen), the maturity of the fruit (Mat) and the age of the callus (Time)) on the amount of rAsA (Table 2). Moreover, the statistical analysis indicated the presence of significant three-way and two-way interactions among all factors under investigation. The interaction Time × Mat had the largest effect size, followed by Time, similarly to the accumulation of AsA.

Collectively, the data denote that the most relevant single factor for the accumulation of total AsA in calli is the time in tissue culture. Moreover, the effect of the time in tissue culture is significantly affected by the interaction with the maturity stage of the fruit used for the explants.

**Figure 4:**
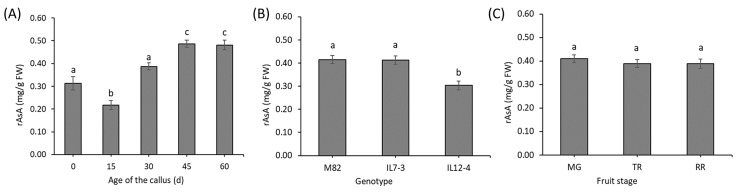
Main effect of the age of the callus (**A**), genotype (**B**) and stage of the fruit used for the tissue culture explants (**C**) on the rAsA content. For each panel, different letters (a–c) represent statistically significant differences (*p* ≤ 0.05).

Post-hoc analysis of the effect of the Time indicated that the age of the callus in tissue culture statistically increased the mean rAsA content (Figure 4A), equally to the AsA content. Moreover, there was a statistical difference between the two ILs (Figure 4B). The main effect of stage of the fruit used as explant was not significant, because, considering all the different times and genotypes, the amount of the mean rAsA was not different (Figure 4C).

### 3.5. Dynamics of Reduced Ascorbic Acid in Tissue Culture

**Figure 5:**
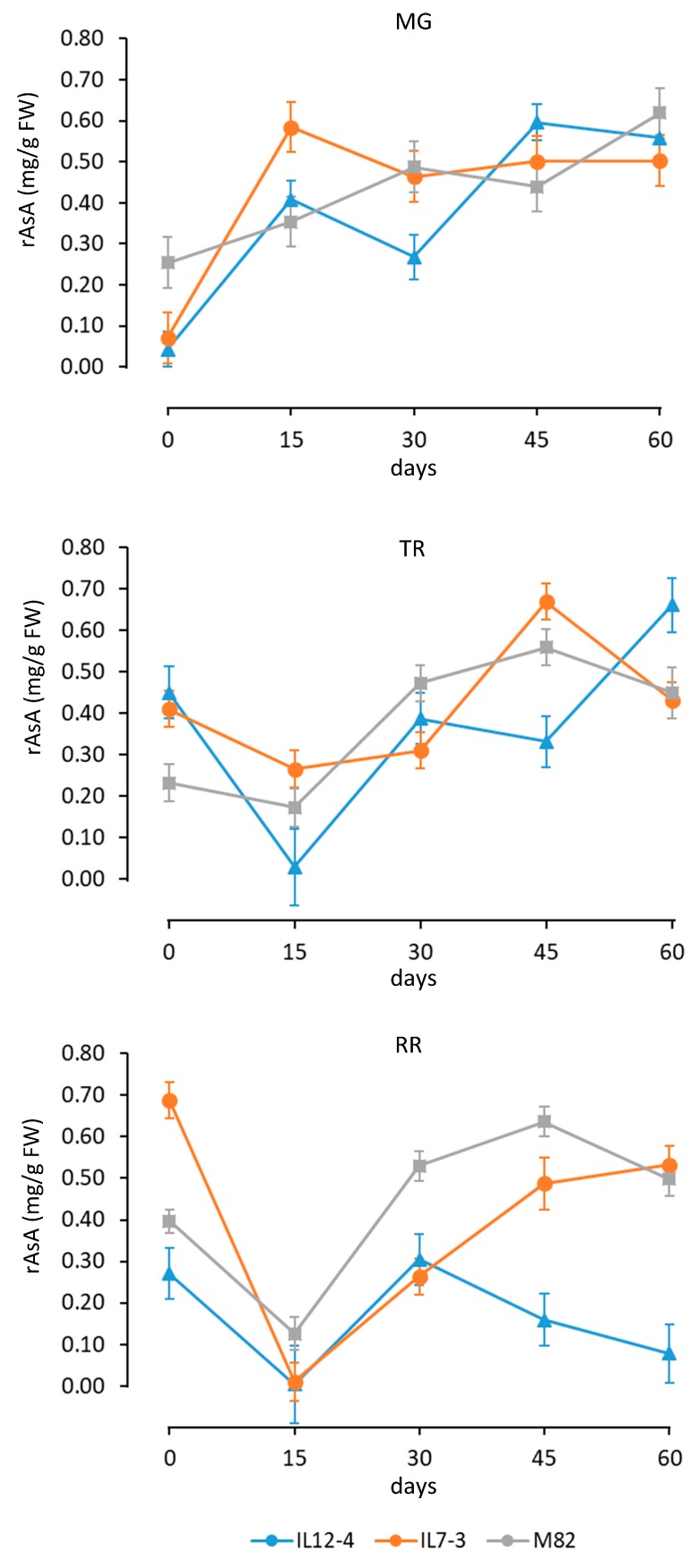
Dynamics of the reduced ascorbic acid (rAsA) in calli deriving from explant of fruits at the mature green (MG), turning (TR), and red ripe (RR) stage. For each source of explants, the graph reports the mean and standard deviation in the different genotypes from 0 to 60 days of tissue culture.

The dynamics of the rAsA accumulation during callus growth for the different sources of explants (fruits at the MG, TR or RR stage) is presented in Figure 5. Differences in rAsA content among the genotypes were less pronounced compared to the AsA. A trend similar to the AsA accumulation was present principally for the MG and RR stages. For the various explants, differences among the genotypes at the latest time point (60 days) were not present, except for the IL12-4 calli deriving from red ripe fruits.

### 3.6. Gene Expression Analysis

Considering that the highest increase in ascorbic acid during the plant tissue culture was observed in calli from mature green explants, we monitored changes in the expression level of genes involved in the AsA biosynthesis in this material. For each genotype, the relative quantification was carried out at the end of the calli cultivation (60 days), setting the expression level at the beginning of the callus cultivation as the calibrator condition.

We analyzed three genes involved in the AsA biosynthesis, namely the GDP-mannose pyrophosphorylase1 (GMP1), GDP-mannose pyrophosphorylase2 (GMP2) and l-galactono-1,4-lactone dehydrogenase (GLDH); five genes involved in the oxidation of l-ascorbic acid, namely the ascorbate oxidase (AO), the cytosolic ascorbate peroxidase 1 (APX1), the cytosolic ascorbate peroxidase 2 (APX2), the peroxisomal ascorbate peroxidase 3 and the stromal ascorbate peroxidase 7 (APX7); and three genes, namely the monodehydroascorbate reductase 1 (MDHAR1), monodehydroascorbate reductase2 (MDHAR2) and dehydroascorbate reductase 1 (DHAR1), coding for enzymes involved in the reduction of ascorbate-oxidised products. In addition, we monitored the expression of a beta-glucosidase (b-GLU) gene as a hint of the hydrolase activity related to plant cell wall metabolism in tissue culture.

**Figure 6:**
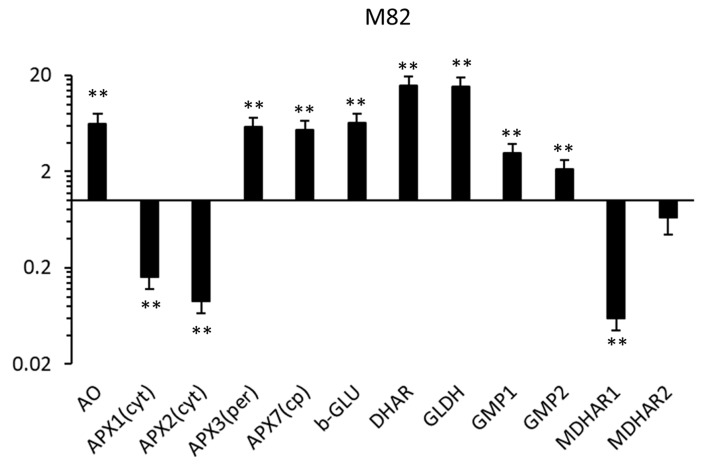

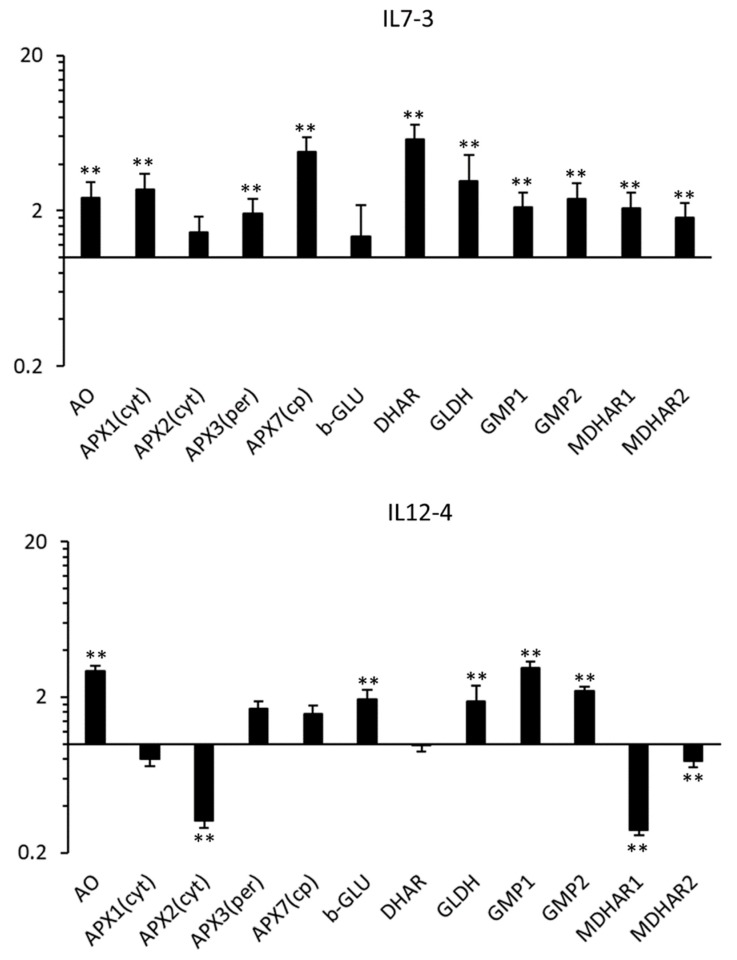
Relative gene expression by real-time PCR of genes involved in AsA biosynthesis. For each genotype (M82, IL7-3 and IL12-4), quantities (RQ) are relative to the calibrator condition (fruit explant) and are graphed on a logarithmic scale. For each gene, asterisks indicate that the 2^−Δ*C*t^ values were significantly different between the test (calli at 60 days) and calibrator condition (** *p* < 0.01; *t*-test).

In all genotypes, the abundance of the transcripts of several genes involved in the ascorbic acid metabolism was significantly altered in calli compared to fruit tissues (Figure 6). The largest difference, both in terms of number of differentially expressed genes (DEGs) and in the RQ values, was in the M82 variety (11 DEGs), followed by the IL 7-3. Moreover, although gene expression is presented as relative quantification (i.e., in relation to a genotype-specific reference condition), the data imply that the effect of the time spent in tissue culture is different for the two ILs. In relation to the calibrator condition (i.e., the fruit explant), eight DEGs were overexpressed in the IL7-3, while four (resp. three) genes were overexpressed (resp. underexpressed) in the IL12-4.

Among the genes involved in AsA biosynthesis, the two GMPs under investigation were overexpressed in the three genotypes. The GLDH was significantly upregulated in M82 and IL7-3 and its relative increase in IL12-4 was just below 2-fold (and not statistically significant). At cellular level, the ascorbic acid pool undergoes a continuous recycling due to a continuous oxidation and reduction. The ascorbate oxidase (AO) gene was another gene overexpressed in the calli of the three genotypes. However, the transcriptional profile of the other genes involved in ascorbate oxidation (i.e., the four ascorbate peroxidases, APXs) was complex, also because the three genotypes showed a different response to the tissue culture.

Considering both the ascorbate peroxidases and the monodehydroascorbate reductases, the M82 and IL12-4 calli showed a similar behavior. Specifically, the downregulation of MDHAR1 associated with the downregulation of the chloroplastic and cytosolic APXs. In IL7-3 calli, the genes involved in ascorbate recycling had a higher level of expression compared to fruit explants. Overall, the expression level of the MDHARs and DHAR under investigation well correlated with that of the cytosolic APXs.

## 4. Discussion

Tomato fruits represent a relevant source of ascorbic acid in the human diet. Previous studies identified two ILs with QTLs for ascorbic acid [16,21,22,23]. In this work, we sought to examine possible differences in the ascorbic acid accumulation among these lines in controlled plant tissue culture conditions, in relation to the maturity stage of the fruit used for the explants and the age of the callus.

Among the genotypes, the relative ability to form callus strongly differed according to fruit ripening stage. While the induction rate was relatively stable in IL7-3, this feature was progressively halved in M82 considering later fruit stages, and strongly reduced in TR and RR explants of the IL12-4. This difference may be related to the ability to recover wounded tissue by producing callus cells in vitro, consistent with the large divergence in wounding response between *S. lycopersicum* and *S. pennelli* [42].

Although calli were recovered with a different efficiency, the data indicate that it is possible to accumulate ascorbic acid from tomato cells cultured in vitro. At the end of our assay, the tAsA content was higher than fruits for the three genotypes and for the different explants, except for those from red ripe fruits. AsA is very unstable (also in media for cell culture), and the progressive increase of AsA amount per fresh weight does not rule out that longer time of callus growth may further enhance its concentration. On the other hand, the narrow and not significant differences between 45 and 60 days imply that a possible gain may be limited.

Several factors influence the accumulation of metabolites in tissue culture conditions [43]. Our work indicated that the genotype, the explant’s maturity stage, and the time in tissue culture significantly affected the AsA content in tomato cells. Moreover, the interactions among factors were also significant. Within this context, it was noteworthy the similarity in AsA content of the three genotypes and of the different explants after 60 days of cultivation. Plant calli cultivated in vitro are “dedifferentiated” cells (that also lack differentiated plastids for photosynthesis) [44], and this feature can explain the limited difference among genotypes after sixty days in tissue culture.

Differences between tAsA accumulation according to the source explants were evident, for instance, after 15 days. Both AsA and rAsA content boosted in the MG explants of the different genotypes. AsA content remains relatively stable at 15 days of tissue culture for TR explant and it showed a reduction for RR explants, although in this instance the effect of the genotype was more evident.

It is generally accepted that morphological differentiation favours the biosynthesis of secondary metabolites, especially those produced in specialized cells [35]. Summing up all the conditions, MG explants produced more tAsA than others yet, this is also due to the increase of both AsA and rAsA at 15 days. It is possible that MG explants more actively reacted to the stress of the initial phase of the tissue culture experiments (e.g., wounding, dehydration, etc.), as also implied by the highest callus induction rate of these explants. Similarly, the IL12-4 RR explants were less amenable to callus induction and accumulated a limited amount of rAsA. However, the relation between stress (such as wounding) and AsA in tomato is complex and not always positive [45,46]. We favour the possibility that the highest accumulation at the early phases of tissue culture of MG explants depends on the active AsA biosynthesis at this maturity stage [47]. Moreover, MG explants are expected to be photosynthetically much more active than other explants and this hypothesis may also account for the reduction of AsA content in RR explants after 15 days.

For the three genotypes, the highest (absolute and relative to the source explant) increase of AsA was observed for the MG explants. To gain some insights in the molecular mechanisms that underline this phenomenon, we quantify the expression of genes involved in AsA metabolism. AsA concentration depends on both transcriptional and post-transcriptional regulatory processes, with transcript level being one of the factors [14,48]. The analysis indicated that genes coding for enzymes that catalyze the first and terminal steps of the Smirnoff-Wheeler pathway for l-ascorbate biosynthesis (such as the two GMPs and the GLDH) were overexpressed (>1.5 fold), consistent with the observed increase of total ascorbic acid. Overall, the M82 expression profile closely resembled the one of the IL12-4, suggesting that in controlled in vitro conditions, IL7-3 has larger differences in the molecular mechanisms for controlling AsA accumulation. Analysis of sub-ILs of 7-3 revealed the presence of several DNA sequences potentially involved in AsA metabolism [49]. An ascorbate peroxidase was overexpressed in all genotypes, although its activation may be multifactorial. For fieldgrown tomato, the relation between AO and AsA content in mature fruits is complex, because transgenic lines with either higher or lower AO activity had higher AsA amount than control lines [50]. In pepper, in addition of a positive correlation between AO activity and AsA level [51], the role of the AO gene has been connected with the regulation of cell division and growth in expanding tissues [52]. In tobacco, the AO expression was also linked to hormone-mediated cell wall loosening [53] and it is well known that auxins stimulate cell elongation also by inducing cell wall loosening. Among the tested genes, a beta-glucosidase, a gene that codes for an enzyme that in vitro conditions is expected to be involved mainly in cell wall loosening [54], was significantly overexpressed in the M82 and IL12-4.

While the production of antioxidants in plant cell culture is well established [55], to our knowledge very limited information is available for the ascorbic acid [56] and other vitamins [57,58]. Our work indicated that in vitro tomato cells can yield AsA well above the amount present in the pericarp of red ripe fruits. Moreover, the data indicated that AsA accumulation in plant cells in vitro depends on various factors and their interaction, with the time of cultivation being positively related to the AsA amount. While the variability among genotypes is significant for the callus induction rate, genetic differences appears to be less relevant with the growth of callus. Contrary to the production of secondary metabolites from specialised cells, the data also indicated that explants from mature green fruits should be preferred. In the future, other factors (e.g., biosynthetic precursors, media, elicitors, etc.) should be optimized for the large-scale production of natural ascorbic acid from plant cells.

## Figures and Tables

**Table 1:** ANOVA summary table for the ascorbic acid.

Source of Variation	Sum of Squares	df	F-Ratio	*p*-Value	Partial η^2^
Time	3.609	4	18.715	<0.001	0.117
Gen	1.321	2	13.702	<0.001	0.046
Mat	0.953	2	9.881	<0.001	0.034
Time × Gen	1.902	8	4.933	<0.001	0.065
Time × Mat	6.146	8	15.936	<0.001	0.184
Gen × Mat	0.812	4	4.214	<0.001	0.029
Time × Gen × Mat	3.899	16	5.392	<0.001	0.125
Error	27.332	618			

**Table 2:** ANOVA summary table for the reduced ascorbic acid.

Source of Variation	Sum of Squares	df	F-Ratio	*p*-Value	Partial η^2^
Time	4.868	4	35.331	<0.001	0.194
Gen	1.186	2	17.221	<0.001	0.055
Mat	0.556	2	8.075	<0.001	0.027
Time × Gen	0.969	8	3.515	0.001	0.046
Time × Mat	5.228	8	18.973	<0.001	0.205
Gen × Mat	1.125	4	8.169	<0.001	0.053
Time × Gen × Mat	3.804	16	6.903	<0.001	0.158
Error	20.288	614			

## Supplementary Materials

The following are available online at https://www.mdpi.com/2076-3921/9/3/222/s1. Table S1: List of the primers used in this study. Primers sequences are in 5′ to 3′ order.
