# Supplementary material for: Accumulation of Ascorbic Acid in Tomato Cell Culture: Influence of the Genotype, Source Explant and Time of In Vitro Cultivation
Source: Antioxidants (Basel). 2020 Mar 7;9(3):222. doi: 10.3390/antiox9030222 (PMC7139686; doi:10.3390/antiox9030222)
Supplement: Supplementary file 1 [file antioxidants-09-00222-s001.pdf]

Supplementary Tables 1: List of the primers used in this study. Primers sequences are in 5’ to 3’ order.

Abbreviation	Name	Accession number	Primer Forward	Primer Reverse
AO	L-ascorbate oxidase	NM_001247900.2	CATGGGCTGATGGAACAGAA	CCCCTGGCACAATAGGACATT
APX1(cyt)	Cytosolic ascorbate peroxidase 1 (APX1)	NM_001247853.2	AGCAGTTTCCCACCCTCTCC	CAACACCAGCCAACTGATGG
APX2(cyt)	Cytosolic ascorbate peroxidase 2 (APX2)	NM_001247859.2	TACGGGAGGACCTGATGTTCC	TCTGGTTTGTCCTCTCTGCCA
APX3(per)	L-ascorbate peroxidase 3 peroxisomal	NM_001308331.1	AGACCTCCGCGCTCTCATC	CGAAGCATGATCGGAGCAC
APX7(cp)	Stromal ascorbate peroxidase 7 (APX7)	NM_001306170.1	ATGGGAGGATGGATGTGTCC	GCCTTCCTTCTTCTGGGCAT
b-GLU	Beta-glucosidase	NM_001247483.2	TGGGGATCAGGATACACACAG	TCATCCAAAGAGCAGAACGCT
DHAR	Dehydroascorbate reductase 1	NM_001247893.2	CCATCTCGAGGTGGCTCTTG	GCTTTCAGGCACACTCCACTT
EF1A	Elongation Factor 1-alpha	NM_001247106.2	GACCAACCCTCCTTGAGGCT	ATGGCCTCTTGGGCTCATTA
GLDH	L-galactono-1,4-lactone dehydrogenase	NM_001247674.3	GATGCATACAATCAAGCGCG	AGATAGGATGCGGTTTGGGTC
GMP1	GDP-D-mannose pyrophosphorylase 1	NM_001247096.2	TTCGGTACTCGGCTCAGGC	TCGACAAGTGGCTTTGGGAC
GMP2	GDP-mannose pyrophosphorylase	XM_004240879.4	GTACCCGGTTGAGGCCATTA	AATCAACCAGTGGCTTTGGG
MDHAR1	Monodehydroascorbate reductase (NADH)-like protein 1	NM_001247084.2	CACGTTTACCGGGCTTTCAT	GTTTGCCTCTCACCACCAGC
MDHAR2	Monodehydroascorbate reductase (NADH)-like protein 2	NM_001331188.1	CACAACTCTATTCAAAGGGCAAG	TGTTTTGAAGAACGCATCTGTC
